# Antibodies against HSV-1 and Curli Show the Highest Correlation in Parkinson’s Disease Patients in Comparison to Healthy Controls

**DOI:** 10.3390/ijms232314816

**Published:** 2022-11-26

**Authors:** Seyedesomaye Jasemi, Kai Paulus, Marta Noli, Elena Rita Simula, Stefano Ruberto, Leonardo A. Sechi

**Affiliations:** 1 Dipartimento di Scienze Biomediche, Università di Sassari, 07100 Sassari, Italy; 2 ASL Sassari, 07100 Sassari, Italy; 3 Struttura Complessa Microbiologia e Virologia, Azienda Ospedaliera Universitaria, 53100 Sassari, Italy

**Keywords:** Parkinson’s disease, humoral response, curli, *P. gingivalis*, *A. actinomycetemcomitans*, Epstein–Barr virus, *M. avium* subspecies *paratuberculosis*, Herpes simplex virus

## Abstract

Parkinson’s disease (PD) is a neurodegenerative disorder involving the accumulation of alpha-synuclein (α-syn)/Lewy bodies in the brain and -enteric nervous system. The etiology of the disease is not well understood, but bacterial and viral infections may contribute to the pathogenesis of PD. It has been suggested that the gastrointestinal (GI) complications observed in PD patients may arise from bacterial dysbiosis, leading to curli/α-syn deposits in the enteric nervous system. Enteric bacteria secrete curli, a functional amyloid peptide involved in adhesion to surfaces, cell invasion, and biofilm formation. However, these bacterial amyloids can initiate additional α-syn deposits through immune system activation and cross-seeding. In this study, we investigate the humoral response against α-syn, curli peptides, and various bacterial and viral immunogen peptides in PD patients, and compare them with those in healthy controls (HCs). Polyclonal IgG antibodies (Abs) were detected against peptides derived from α-syn (α-syn_100–114_), curli (Curli_133–141_), *Porphyromonas gingivalis* Pg (RgpA_800–812_, Kpg_328–339_), *Aggregatibacter actinomycetemcomitans* (LtxA1_429–445_, LtxA2_64–80_), *Mycobacterium avium* subsp. *paratuberculosis* (MAP3865c_125–133_, MAP1,4-a-gbp_157–173_ and MAP_4027_18–32_), Epstein–Barr virus (EBNA1_400–413_, BOLF1_305–320_), and Herpes Simplex virus 1 (UI42_22–36_), as investigated by indirect ELISA of 51 serum samples from PD and 58 sex and age-matched HCs. Significant differences in OD (optical density) values and Abs positivity between PD patients and HCs were observed for Kpg (82.3% vs. 10.3%), followed by RgpA (60.7% vs. 24.1%), curli (51% vs. 22.4%), and UI42 (43.1% vs. 25.8%) in PD, compared to HCs sera (*p* < 0.001). No significant difference was found in the ODs obtained from other tested peptides in PD patients, compared to HCs. Significant positive correlations between OD values obtained by ELISA were observed for UI42 and curli (*r* = 0.811, *p* < 0.0001), Kpg and RgpA (*r* = 0.659, *p* < 0.0001), followed by LtxA1 and LtxA2 (*r* = 0.653, *p* < 0.0001). The correlation between the HY scale (Hoehn and Yahr Scale) and LtxA1 (*r* = 0.306, *p* < 0.028) and HY and Kpg (*r* = 0.290, *p* < 0.038) were significantly positive. This study reports a significantly increased humoral response against curli, Pg, and HSV-1 in PD patients, implying that they could be important factors in the pathogenesis of the disease. In addition, the high positive correlation between UI42 and curli may suggest the involvement of HSV-1 in GI dysbiosis. Therefore, the role of each individual pathogen and curli in PD needs to be further investigated.

## 1. Introduction

Parkinson’s disease (PD) is the second-most common neurodegenerative disease, with a prevalence of more than 1% in the population above 65 years of age which is projected to double by 2030 [1]. PD patients frequently suffer from motor dysfunctions, including resting tremors, bradykinesia, rigidity, and gait abnormalities, as well as non-motor symptoms such as hyposmia, sleep disorders, depression, cognitive impairment (CI), and gastrointestinal (GI) symptoms [2,3,4,5].

PD is a multifactorial disorder resulting from a combination of genetic and environmental factors [5,6]. Over the past decade, it has become increasingly apparent that the gut microbiota and microbial pathogens may contribute to neurodegenerative diseases, either directly or through immune activation [7]. A recent study has shown that curli-producing bacteria in the gut microbiota could promote alpha-synuclein (α-syn) aggregation in both the GI tract and the brain, through immune system activation and/or cross-seeding (heterologous amyloid curli protein acts as a seed and facilitate the aggregation of α-syn) [7]. Thus, several bacteria and viruses have been associated with PD, including Herpes Simplex Virus 1 (HSV-1), *Porphyromonas gingivalis* (Pg), *Mycobacterium avium* subsp. *paratuberculosis* (MAP), Epstein–Barr virus (EBV), Cytomegalovirus (CMV), *Helicobacter pylori* (*H*. *pylori*), and *Chlamydia pneumoniae* (*C. pneumoniae*) [3,8,9,10,11].

The neuropathological changes in PD are defined by the degeneration of dopaminergic neurons, with the accumulation and aggregation of human amyloid protein α-syn to form Lewy bodies (LBs) in the brain and nervous system [12]. Moreover, α-syn deposits in the brain and enteric nervous system mediated by bacterial dysbiosis may happen years before the disease [13,14]. Interesting evidence has shown that the microbial amyloid curli is structurally similar to human amyloid α-syn, which is involved in PD [15]. Curli makes up as much as 85% of the extracellular matrix of enteric biofilms, which frequently contribute to cell–cell attachment and bacterial invasion in GI biofilms. This protein is expressed when enteric bacteria are grown under stressful environmental conditions [4,15].

The immune system recognizes both the bacterial amyloid curli and human amyloid utilizing the same receptors, such that bacterial amyloid also stimulates the immune system and induces inflammation [16,17] however, these bacterial amyloids can initiate additional α-syn deposits through cross-seeding, potentially indirectly causing neuroinflammation and then neurodegeneration [15].

A comprehensive demonstration of the role of the humoral immune response against bacterial amyloid and multiple pathogens in the PD pathogenesis is still lacking. Therefore, in this study, we aimed to evaluate the prevalence and magnitude of the immune response against immunogen peptides derived from human α-syn_100–114_ and bacterial amyloid curli (Curli_133–141_), *Porphyromonas gingivalis,* Pg (RgpA_800–812_, Kpg_328–339_), *Aggregatibacter actinomycetemcomitans*, Aa (LtxA1_429–445_, LtxA2_64–80_), *Mycobacterium avium* subsp. *paratuberculosis* (MAP3865c _125–133,_ MAP1,4-a-gbp_157–173_ and MAP_4027_18–32_), Epstein–Barr virus (EBNA1_400–413_, BOLF_1305–320_), and Herpes Simplex Virus HSV-1 (UI42_22–36_) in PD patients compared with the general population.

## 2. Results

For this case–control study, we investigated a set of serum samples derived from 51 PD patients (27 females, 24 males; median ± SD: 74.05 ± 8.6) and compared their results with 58 HCs (30 females, 28 males; median ± SD: 72.5 ± 8.5). We found no statistically significant difference between the age and sex of PD patients compared to HCs (*p* = 0.346 and *p* > 0.99, respectively).

**Figure 1:**
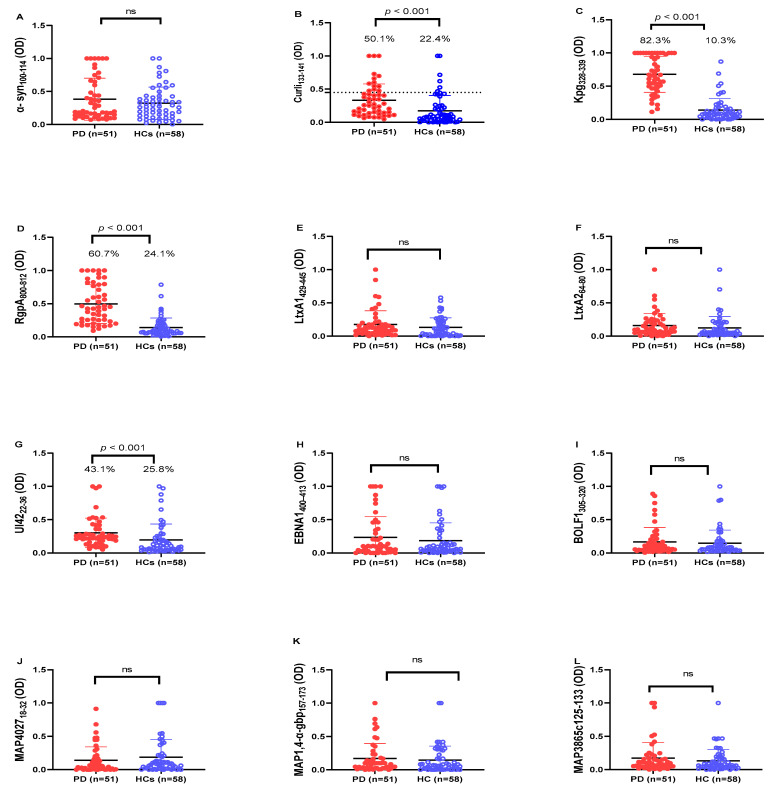
Analysis of Abs reactivity against human α-syn and pathogenic microorganism-derived peptides in PD patients and HCs. Serum samples were tested with indirect ELISA assay to plate-coated (**A**) α-syn_100–114_, (**B**) Curli _133–141_, (**C**) Kpg_328–339_, (**D**) RgpA_800–812_, (**E**) LtxA1_429–445_, (**F**) LtxA2_64–80_, (**G**) UI42_22–36_, (**H**) EBNA1_400–413_, (**I**) BOLF_1305–320_, (**J**) MAP4027_18–32_, (**K**) MAP1,4-α-gbp_157–173_, (**L**) MAP3865c_125–133_ peptides. The dotted lines represent the cut-off values calculated by ROC analysis; In the upper section of the graph, are indicated the Mann–Whitney *p*-value and the percentage of positive patients’ values calculated by the Fisher’s exact test. Statistical significant levels showed at *p* < 0.05, ns: not significant.

The intra- and inter-assay variation were 8.2–9.9% and 12.1–14.7%, respectively. Optical density values (OD) at 405 nm (OD405) were obtained from the IgG indirect-ELISA protocol with respect to 12 designed immunogenic peptides for all PD and HC sera, as demonstrated in Figure 1.

Based on our results, antibodies (Abs) against human amyloid-derived peptide (α-syn_100–114_) did not significantly different between PD patients and HCs (Figure 1A; *p* > 0.05), whereas Abs level against bacterial amyloid-derived peptide (Curli_133–141_) was significantly higher in PD patients (50.1%; 26 out of 51) than in HCs (22.4%; 13 out of 58) (cut-off value 0.45; AUC = 0.756; *p* < 0.0001; see Figure 1B).

In addition, we observed a remarkable Abs response against the microorganism-derived peptides Pg (Kpg_328–339_, RgpA_800–812_,) and HSV-1 (UI42_22–36_) in the sera of PD patients, which was significantly higher when compared to HCs. For Kpg_328–339_, we observed a significant difference in the Abs response between PD and HCs (Figure 1C). In total, 82.3% (42 out of 51) of PD patients showed a positive Abs response against Kpg peptide, whereas just 10.3% (6 out of 58) were positive in the HC group (cut-off value 0.39; AUC = 0.95; *p* < 0.0001; see Figure 1C). Concerning RgpA_800–812_ peptide, we observed a significantly higher Abs response in PD patients (60.7%; 31 out of 51) than in HCs (24.1%; 14 out of 58) (cut-off value 0.29; AUC = 0.902; *p* < 0.0001; see Figure 1D). The positivity and mean levels of anti- UI42_22–36_ Abs also showed a significant difference between PD patients (43.1%; 22 out of 51) than HCs (25.8%; 15 out of 58) (cut-off value 0.25; AUC = 0.741; *p* < 0.0001; see Figure 1G).

The level of Abs against other immunogenic peptides (LtxA1_429–445_, LtxA2_64–80_, EBNA1_400–413_, BOLF_1305–320_, MAP4027_18–32_, MAP1,4-α-gbp_157–173_, MAP3865c_125–133_ peptides) did not statistically differ between PD patients and HCs (*p* > 0.05).

**Figure 2:**
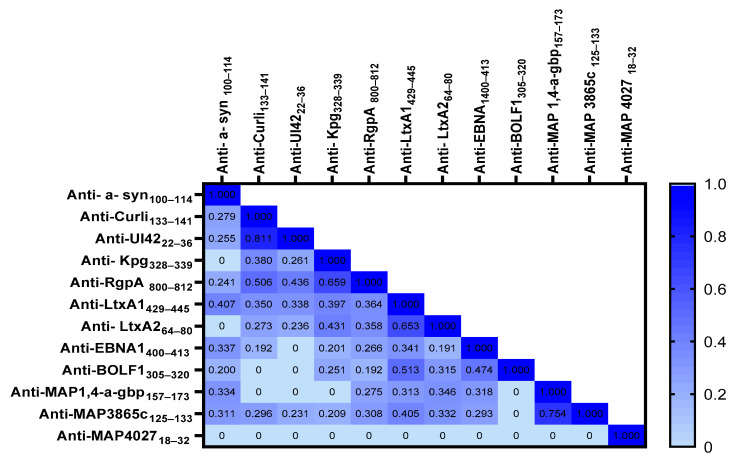
Heatmap shows the *r* values obtained from Spearman correlation analysis performed among derived peptides’ ODs.

Spearman’s correlation analysis was conducted to determine the possible correlations between OD values obtained against different immunogenic peptides between PD patients and HCs. The highest rate of correlation was observed between anti-UI42_22–36_ and anti- Curli _133–141_ (r = 0.811, *p* < 0.0001), followed by anti-RgpA_800–812_ and anti-Kpg_328–339_ (r = 0.659, *p* < 0.0001), anti-LtxA1_429–445_ and anti-LtxA2_64–80_ (r = 0.653, *p* < 0.0001), anti-BOLF1_305–320_ and anti-LtxA1_429–445_ (r = 0.513, *p* < 0.0001), and anti-Curli _133–141_ and anti-RgpA _800–812_ (r = 0.506, *p* < 0.0001). Figure 2 shows the r values obtained from the Spearman correlation analysis performed among the derived OD against the designed peptides.

In addition, a further Spearman correlation analysis was performed to evaluate a possible correlation between the severity of disease (HY scale 1 to 5) and ODs values derived from the indirect-ELISA assay. There were significant correlations between ODs against LtxA1_429–445_ and the HY scale (*r* = 0.306, *p* < 0.028), and between Kpg_328–339_ and the HY scale (*r* = 0.290, *p* < 0.038) in PD sera. There was no significant correlation between the OD values against other peptides and the HY scale.

## 3. Discussion

Parkinson’s Disease (PD) is a complex neurodegenerative amyloid disorder with unknown cause [1,6]. Growing evidence has demonstrated that the gut microbiota and microbial pathogens are involved in its etiology [7,18]. In addition, novel findings have emphasized that amyloid curli produced by Gram-negative enteric bacteria in the biofilm state in the GI tract has a link to neurodegenerative diseases [15].

In this study, Abs against human amyloid α-syn and bacterial amyloid curli were investigated. We observed, for the first time, an increase in Abs level against bacterial amyloid curli in PD patients, compared to HCs (*p* < 0.005), while no significant difference was observed for anti-human amyloid between the two groups. Several studies have reported no difference in serum human amyloid α-syn Abs between patients with PD and HCs [19,20], consistent with our study. In contrast, several other studies have found high levels of α-syn Abs in PD patients, compared to HC sera [21,22]. Future investigations are necessary to determine the α-syn Abs level sub-classes in the different stages of PD, compared with HC for use as a therapeutic or diagnostic biomarker in PD patients.

Interestingly, in our study, the Abs against bacterial amyloid curli was significantly higher in PD patients than in HCs. The presence of Abs to a key biofilm component curli in 51% PD vs. 22.4% of HCs suggests that biofilm may have a potential role in the development of PD, possibly as cryptic reservoirs of α-syn homolog curli. A recent finding has demonstrated that the presence of curli-expressing *E. coli* in mice microbiota increases α-syn-mediated motor deficits and brain pathology [23]. A considerable correlation between persistent bacteriuria and anti-curli/eDNA IgG levels (IgGs against curli naturally complexed with bacterial extracellular DNA), detected in lupus and HC plasma, has been described in the study of Pachucki et al. [24]. In addition, IgA anti-curli/eDNA levels were higher in lupus donors, compared to controls [24]. However, detailed knowledge regarding the role of curli in the stimulation of the immune system and its relationship with PD requires further investigation. Moreover, this is the first report of anti-curli IgGs in PD patients, which could be a promising target for treatment and as a diagnostic biomarker in PD. Furthermore, we observed a higher prevalence of humoral response against peptides derived from periodontal pathogens Pg and Aa in PD patients, compared to HCs, which was statistically meaningful for the anti-RgpA and Kpg IgG peptides. Periodontal pathologies are known to be linked to systemic inflammation [25], and *P. gingivalis* (Pg), especially, is associated with different systemic diseases, including PD, non-insulin-dependent diabetes mellitus [26,27], Alzheimer’s disease [28], rheumatoid arthritis [29,30], and cardiovascular disease [31,32]. These findings indicate the possible association between Pg and PD, confirming the hypothesis that Pg can induce a systemic antibody response, possibly influencing the progression of PD. On the other hand, there was a significant relationship between the increase in the level of antibodies against this bacterium and the severity of Parkinson’s disease (HY index). This relationship emphasizes the role played by oral infection during Parkinson’s disease. Despite various studies on the relationship of Pg with PD, no study was found on the relationship of Aa with PD. Díaz-Zúñiga et al. study showed that Aa can increase the risk of Alzheimer’s disease by specific inflammatory and immune responses in brain cells [33]. Therefore, the association between periodontal pathogens, especially Aa, in the progression of neurodegenerative diseases thus needs to be further investigated. It also seems that accurate oral and dental hygiene in Parkinson’s patients can be effective in prevention and reduction the symptoms of the disease. We observed a statistically significant difference in antibody levels against a common pathogen of the central nervous system, HSV-1 (Ul42_22–36_) in PD patients in comparison to HCs [9], which was consistent with other studies [ 34]. Furthermore, the antibodies able to recognize the HSV-1-Ul42_22–36_ peptide are able to cross-react with the homologous human α-syn_100–114_ epitope [9]. In this study, we highlight a positive correlation between Abs against HSV-1 and curli, supporting the hypothesis that HSV-1 infection may change the composition of the gut microbiota, which may lead to dysbiosis. The results from the study of Ramakrishna et al. showed that HSV and acyclovir can disrupt the gut bacterial community in a sex-biased manner in a C57BL/6 mice model [35]. In our study, the level of Abs against EBV peptides was higher in PD patients than in the healthy group; however, this difference was not statistically significant. Epidemiological studies have demonstrated that PD patients are significantly more Abs seropositive for EBV than HCs [36,37]. Latent EBV infection can trigger autoantibodies that can cross-react with α-syn and elevate α-syn aggregation [36,37]. Considering that EBV is one of several proposed environmental factors associated with PD, our study population is probably influenced by other genetic and environmental factors. On the other hand, to obtain more accurate epidemiological statistical results, it is suggested to investigate of Abs against other EBV-immunogenic peptides in a larger number of PD patients and comparison with HC, in future studies. As has been reported in a previous study, a high level of Ab-mediated immune reaction was detected against MAP3865c_207–219,_ and MAP3865c_82–97_ peptides, while no significant reaction was observed against MAP3865c_81–95_ and MAP3865c_44–59_ peptides [8]. In this study, Abs levels against other MAP epitopes were the same in the two populations (i.e., PD and HCs).

The most obvious limitation of the current study was that of small sample size for the evaluation of Abs against these peptides. Moreover, selection of other microbial peptides with broad immunogenic potential designed from these organisms and checking Abs against them in large sample sizes is recommended.

## 4. Materials and Methods

### 4.1. Study Population and Blood Collection

For the current case–control study, we examined two populations, PD patients, and healthy controls (HCs) during the period between July 2021 and August 2022. This study was approved by the Ethics Committee of the University of Sassari in 2019 (prot 2159/CE). Informed consent was obtained from all individual participants. All patients were diagnosed based on medical history, clinical symptoms and neurological and physical examination. All data on age, gender, and HY scale (Hoehn and Yahr Scale) were retrieved from patient records. During the same period, healthy controls, with no personal or familial history of diagnostic PD, whose age and gender matched with those of the patients, were included in the study as controls. Blood samples were collected from participants referred to the Parkinson Institute hospital at the Azienda Ospedaliera Universitaria of Sassari. Then, sera were separated according to the standard method [38] and preserved at –80 °C in a freezer.

### 4.2. Peptides

Synthesis of an immunogenic peptide derived from bacterial amyloid curli (Curli_133–141_: NSSVNVTQV) was designed using the Immune Epitope Database and Analysis Resource (IEBD) and synthesized at >95% purity (LifeTein, South Plainfield, NJ, USA).

Immunogenic peptides derived from human amyloid (α-syn_100–114_), *Porphyromonas gingivalis*, Pg (RgpA_800–812_, Kpg_328–339_), *Aggregatibacter actinomycetemcomitans* (LtxA1_429–445_, LtxA2_64–80_), *Mycobacterium avium* subsp. *paratuberculosis* (MAP3865c_125–133,_ MAP1,4-a-gbp_157–173_ and MAP_4027_18–32_), Epstein–Barr virus (EBNA1_400–413_, BOLF_1305–320_), and Herpes Simplex Virus 1 (UI42_22–36_) were selected from peptides used in previous studies [9,18,30,39]. All peptides were re-suspended in dimethyl sulfoxide (DMSO) at a final concentration of 10 µg/mL and stored at −80 ◦C until further use (Table 1).

### 4.3. Enzyme-Linked Immunosorbent Assay (ELISA)

Indirect ELISA was performed to investigate the specific IgG antibodies against the designed peptides mentioned in the study. In brief, 50 µL of each peptide at a concentration of 10 µg/mL in 0.05 *M carbonate*/bicarbonate buffer, at pH 9.5 (Sigma-Aldrich, St. Louis, MO, USA), were coated in 96-well plates (Thermo Fisher Scientific, South San Francisco, CA, USA) and incubated at 4 °C for 1 day. The coating solution on plates was removed and blotted on paper towels. Plates were incubated for one hour at room temperature (RT) in a blocking solution with 200 µL of 5% non-fat dried milk (5 g non-fat dried milk powder in 100 mL 1× PBS; Sigma-Aldrich, St. Louis, MO, USA) and washed twice in a solution with 0.05% Tween-20 and phosphate-baffered saline (1× PBS-T; Sigma-Aldrich, St. Louis, MO, USA). Plasma samples (diluted 1:100; 1 µL plasma to 99 µL 1× PBS-T) were added, and the plates were incubated for 2 h at RT. Then, each plate was washed five times in 1× PBS-T and incubated for one hour at RT with 100 µL of PBS and anti-human IgG alkaline phosphatase conjugated antibody produced in goat (1:1000; Sigma-Aldrich, St. Louis, MO, USA). Plates were washed five times in 1× PBS-T and incubated in a dark environment for eight to ten minutes in milli-Q water and p-nitrophenyl phosphate (One p-290 nitrophenyl phosphate tablet and one Tris buffer tablet were dissolved in 20 µL of milli-Q water; Sigma-Aldrich, St. Louis, MO, USA), and the optical density (OD) was read at a wavelength of 405 nm using a microplate reader (Molecular Devices, Sunnyvale, CA, USA). All samples were repeated in duplicate, and positive controls were used for each peptide. The positive sample was a sample previously tested for strong reactivity to the selected peptides, and not reactivity to irrelevant peptides, in order to verify the binding specificity. The negative controls were samples from patients previously tested for the same peptides which had a weak reaction. Moreover, a technical negative control was added, where no sera was added into the peptide-coated wells. The OD values were normalized to a highly positive control serum with absorbance reactivity set at 1.0 OD. Results are expressed as means of duplicate 405 nm OD values.

Intra-assay variation was calculated based on the mean of the CV percentages (%CVs) obtained from OD measurements repeated two times for each serum in the same plate. Inter-assay variation was calculated based on the mean of %CVs obtained from experiments repeated two times for each serum in two separate plates on two different days. Inter-assay variation was done for 30 serum samples with high, low, and moderate ODs.

### 4.4. Statistical Analysis

The analysis was performed using GraphPad Prism version 8.0 software (San Diego, CA, USA). The data distribution was analyzed using the D’Agostino–Pearson omnibus normality test and, consequently, the Shapiro–Wilk test. Non-parametric data were analyzed using the Mann–Whitney U test to compare Abs against different peptides in PD patients compared to HCs. Student’s t-test and Fisher’s exact test were applied to compare the matching of age and sex in PD patients with the HCs group. A value of *p* < 0.05 was considered significant. Optimal cut-off points to discriminate between positive and negative samples were identified based on the receiver operating characteristic (ROC) curve with ≥90% specificity and 95% confidence interval. In addition, Fisher’s exact test was employed to compare the percentages of positive subjects in the two groups. The correlation between OD values obtained by the ELISA test from different peptides was explored through bivariate correlation and regression analysis using the Stata software. In addition, the correlation analysis between the HY scale and OD values obtained by the ELISA test from different peptides was explored through bivariate correlation and regression analysis. 

## 5. Conclusions

In this study, we reported a significantly increased humoral response against curli, Pg, and HSV-1 in PD patients, thus implying the important role of these factors in the pathogenesis of the disease. Therefore, while the development of PD has not yet been associated with unique microbial species and their products, more studies will be necessary to examine the potential interactions between the bacterial amyloid curli and the human amyloid, in order to understand their relevance in the pathogenesis of PD. In addition, A better comprehension of the intricate relationship between microbial pathogens and PD may help in the future to develop effective strategies to detection and preventing the development of the PD.

## Figures and Tables

**Table 1:** Immunogenic peptides used as antigens in the ELISA assay.

	Epitope Sequence	Epitope Position
α-syn_100–114_	LGKNEEGAPQEGILE	100–114
Curli_133–141_	NSSVNVTQV	133–141
UI42_22–36_	LGQPEEGAPCQVVLQ	22–36
RgpA_800–812_	ADPVVTTQNIIVT	800–812
Kpg_328–339_	VTDLYYSAVDGD	328–339
LtxA1_429–445_	AWENKYGKNTFENGYDA	429–445
LtxA2_64–80_	TALIKAAQKLGIEVYHE	64–80
MAP3865c_125–133_	MIAVALAGL	125–133
MAP1,4-a-gbp_157–173_	GTVELLGGPLAHPFQPL	157–173
MAP_4027_18–32_	AVVPVLAYAAARLL	18–32
EBNA1_400–413_	PGRRPFFHPVGEAD	400–413
BOLF1_305–320_	AAVPVLAFDAARLRLLE	305–320

## Data Availability

The data that support the finding of this study are available from the corresponding author, upon reasonable request.
